# Crystal structures of two dis-symmetric di-Schiff base compounds: 2-({(*E*)-2-[(*E*)-2,6-di­chloro­benzyl­idene]hydrazin-1-yl­idene}meth­yl)-6-meth­oxy­phenol and 4-bromo-2-({(*E*)-2-[(*E*)-2,6-di­chloro­benzyl­idene]hydrazin-1-yl­idene}meth­yl)phenol

**DOI:** 10.1107/S2056989020006416

**Published:** 2020-05-19

**Authors:** Rohit B. Manawar, Chandankumar T. Pashavan, Manish K. Shah, Mukesh M. Jotani, Edward R. T. Tiekink

**Affiliations:** a Chemical Research Laboratory, Department of Chemistry Saurashtra University, Rajkot - 360005, Gujarat, India; b Department of Physics, Bhavan’s Sheth R. A. College of Science, Ahmedabad, Gujarat 380001, India; c Research Centre for Crystalline Materials, School of Science and Technology, Sunway University, 47500 Bandar Sunway, Selangor Darul Ehsan, Malaysia

**Keywords:** crystal structure, Schiff base, Hirshfeld surface analysis, computational chemistry

## Abstract

Each of the title dis-symmetric di-Schiff base compounds, C_15_H_12_Cl_2_N_2_O_2_ (I) and C_14_H_9_BrCl_2_N_2_O (II), features a central azo-N—N bond connecting two imine groups, each with an *E*-configuration. One imine bond in each mol­ecule connects to a 2,6-di­chloro­benzene substituent while the other links a 2-hydroxyl-3-meth­oxy-substituted benzene ring in (I) or a 2-hydroxyl-4-bromo benzene ring in (II). Each mol­ecule features an intra­molecular hydroxyl-O—H⋯N(imine) hydrogen bond. The C—N—N—C torsion angles of −151.0 (3)° for (I) and 177.8 (6)° (II) indicates a significant twist in the former. The common feature of the mol­ecular packing is the formation of supra­molecular chains. In (I), the linear chains are aligned along the *a*-axis direction and the mol­ecules are linked by meth­oxy-C—H⋯O(meth­oxy) and chloro­benzene-C—Cl⋯π(chlorobenzene) inter­actions. The chain in (II) is also aligned along the *a* axis but, has a zigzag topology and is sustained by Br⋯O [3.132 (4) Å] secondary bonding inter­actions. In each crystal, the chains pack without directional inter­actions between them. The non-covalent inter­actions are delineated in the study of the calculated Hirshfeld surfaces. Dispersion forces make the most significant contributions to the identified inter­molecular inter­actions in each of (I) and (II).

## Abstract 2

An *E*-configuration about the imine bond is found in both the title mol­ecules, which differ in their central C—N—N—C torsion angles. The main feature of the mol­ecular packing in both crystals is the formation of supra­molecular chains: the linear chains in (I) are consolidated by meth­oxy-C—H⋯O(meth­oxy) and chloro­benzene-C—Cl⋯π(chloro­benzene) inter­actions while the zigzag chains in (II) are sustained by Br⋯O secondary bonding inter­actions.

## Chemical context   

Schiff base mol­ecules, known for their ease of formation, can be deprotonated to form a prominent class of multidentate ligands for a full range of metal ions leading to a rich coordination chemistry (Vigato & Tamburini, 2004 ▸; Clarke & Storr, 2014 ▸). The broad range of biological activities exhibited by Schiff base mol­ecules such as anti-bacterial, anti-viral, anti-fungal, anti-malarial, anti-inflammatory, *etc*. (Naeimi *et al.*, 2013 ▸; Mukherjee *et al.*, 2013 ▸) is a key motivation for studies in this area. Indeed, this is the motivation for the preparation of dis-symmetric di-Schiff base mol­ecules (Liu *et al.*, 2018 ▸) related to the title compounds and their transition-metal complexes (Manawar *et al.*, 2019*a* ▸), complemented by crystallographic studies (Manawar *et al.*, 2019*b* ▸, 2020 ▸). In a continuation of these structural studies, the crystal and mol­ecular structures of meth­oxy- (I) and bromine-substituted (II) analogues of an earlier published dis-symmetric di-Schiff base (Manawar *et al.*, 2019*b* ▸) are described herein, together with the detailed analysis of the mol­ecular packing by Hirshfeld surface analysis and computation of energy frameworks.
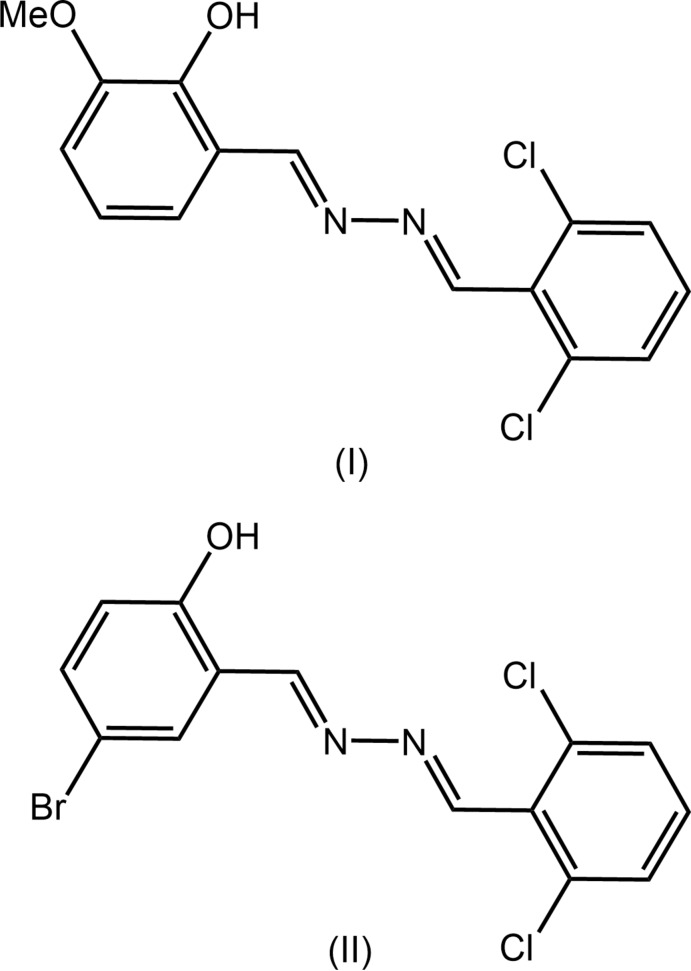


## Structural commentary   

**Figure 1:**
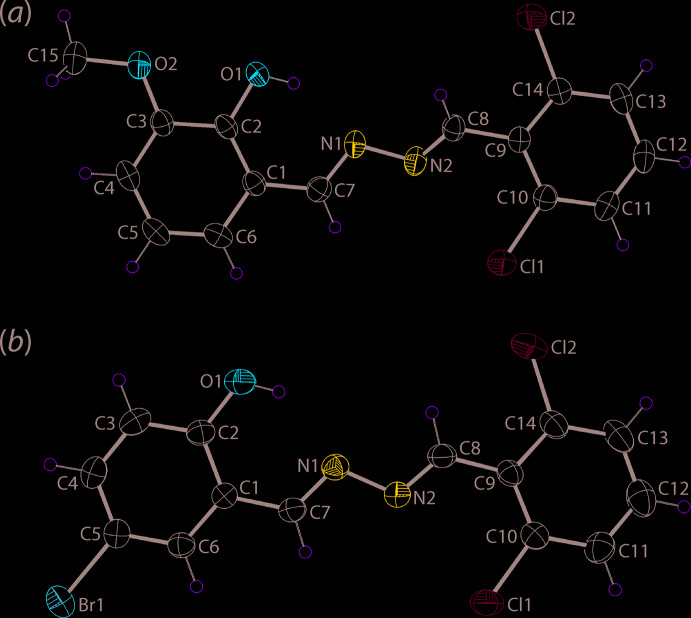
The mol­ecular structures of (*a*) (I) and (*b*) (II), showing the atom-labelling schemes and displacement ellipsoids at the 35% probability level.

The mol­ecular structures of (I) and (II) are shown in Fig. 1 ▸. The common feature of each mol­ecule is the presence of two imine bonds connected by a azo-N—N bond, Table 1 ▸. At one end of each mol­ecule is a 2,6-di­chloro­benzene substituent. In (I), the mol­ecule is terminated by a 2-hydroxyl-3-meth­oxy-substituted benzene ring and in (II), the terminal group is a 2-hydroxyl-4-bromo benzene ring. The configuration about each of the imine bonds is *E*. Each mol­ecule features an intra­molecular hydroxyl-O—H⋯N(imine) hydrogen bond with geometric details listed in Tables 2 ▸ and 3 ▸, respectively. As might be expected and judged from the data in Table 1 ▸, there is a close similarity in comparable geometric parameters characterizing mol­ecules (I) and (II) with salient bond lengths being equal within experimental error. The most significant difference in bond angles is seen in the *ca* 3° wider C9—C8—N2 angle in (II) *cf*. (I). There is an apparent difference in conformation in the central region of the mol­ecules as seen in the *ca* 25° difference in the C7—N1—N2—C8 torsion angles indicating a discernible kink in (I). The central C_2_N_2_ chromophore in (I) exhibits distortions from co-planarity as the r.m.s. deviation of the fitted atoms is 0.1459 Å with maximum deviations to either side of the plane being 0.155 (17) Å for the N2 atom and 0.149 (14) Å for C8. By contrast, the r.m.s. deviation for the central atoms in (II) is 0.0112 Å. Further differences are noted in dihedral angles between the central plane and pendant benzene rings, and between the benzene rings, Table 1 ▸, with the maximum difference occurring for the (C7,N1,N2,C8)/(C9–C14) dihedral angles of 23.1 (4) and 1.5 (6)° for (I) and (II), respectively.

## Supra­molecular features   

**Figure 2:**
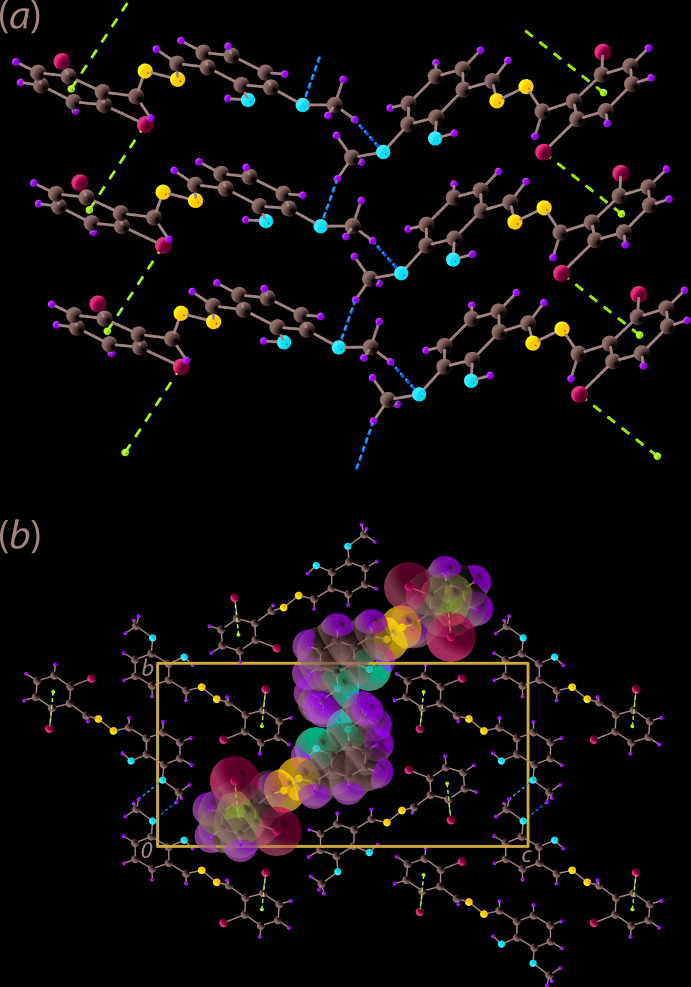
Mol­ecular packing in the crystal of (I): (*a*) supra­molecular chain sustained by meth­oxy-C—H⋯O(meth­oxy) and chloro­benzene-C—Cl⋯π(chloro­benzene) inter­actions shown as orange and purple dashed lines, respectively and (*b*) a view of the unit-cell contents in projection down the *a* axis with one chain highlighted in space-filling mode.

The two prominent directional inter­actions in the mol­ecular packing of (I) are of the type C—H⋯O and C—Cl⋯π, Table 2 ▸. Thus, meth­oxy-C—H⋯O(meth­oxy) and chloro­benzene-C—Cl⋯π(chloro­benzene) contacts serve to link mol­ecules into supra­molecular chain aligned along the *a*-axis direction, Fig. 2 ▸(*a*). The linear chains thus formed assemble in the crystal without directional contacts between them, Fig. 2 ▸(*b*).

**Figure 3:**
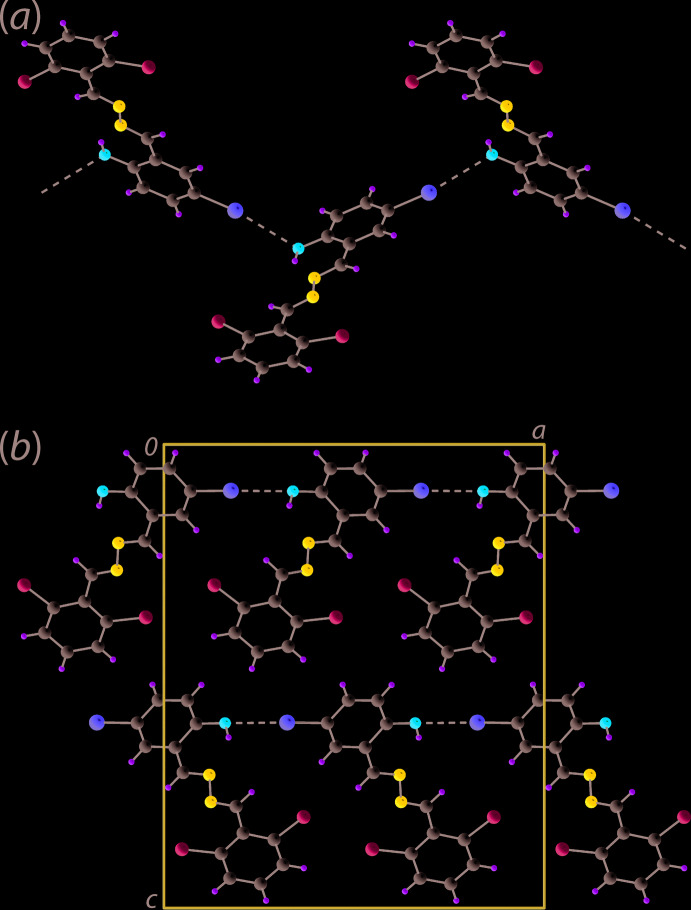
Mol­ecular packing in the crystal of (II): (*a*) supra­molecular, zigzag chain sustained by Br⋯O secondary bonding inter­actions shown as black dashed lines and (*b*) a view of the unit-cell contents in projection down the *b* axis.

Supra­molecular chains along the *a* axis are also noted in the packing of (II), Fig. 3 ▸(*a*). In this instance, the contacts between mol­ecules are of the type Br⋯O, *i.*e. the Br1⋯O1 separation is 3.132 (4) Å for symmetry operation 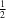 + *x*, 3 − *y*, *z*. With the first such inter­action in a crystal being reported in 1954, *i.e*. in the crystal of Br_2_·O(CH_2_CH_2_)_2_O (Hassel & Hvoslef, 1954 ▸), these well-described secondary bonding inter­actions (Alcock, 1972 ▸), are termed halogen-bonding inter­actions in the current parlance (Tiekink, 2017 ▸). In (II), the Br⋯O inter­actions assemble mol­ecules into zigzag chains as these are propagated by glide symmetry. Globally, the supra­molecular chains stack along the *b* axis to form layers and the layers stack along the *c* axis in an …*ABAB*… fashion, Fig. 3 ▸(*b*), but there are no directional inter­actions between the chains.

## Hirshfeld surface analysis   

The Hirshfeld surfaces for (I) and (II) were calculated employing the *Crystal Explorer 17* program (Turner *et al.*, 2017 ▸) following recently published protocols (Tan *et al.*, 2019 ▸). The results describe the influence of non-bonded inter­actions upon the mol­ecular packing in the crystals of (I) and (II), especially in the absence of directional inter­actions between the chains.

**Figure 4:**
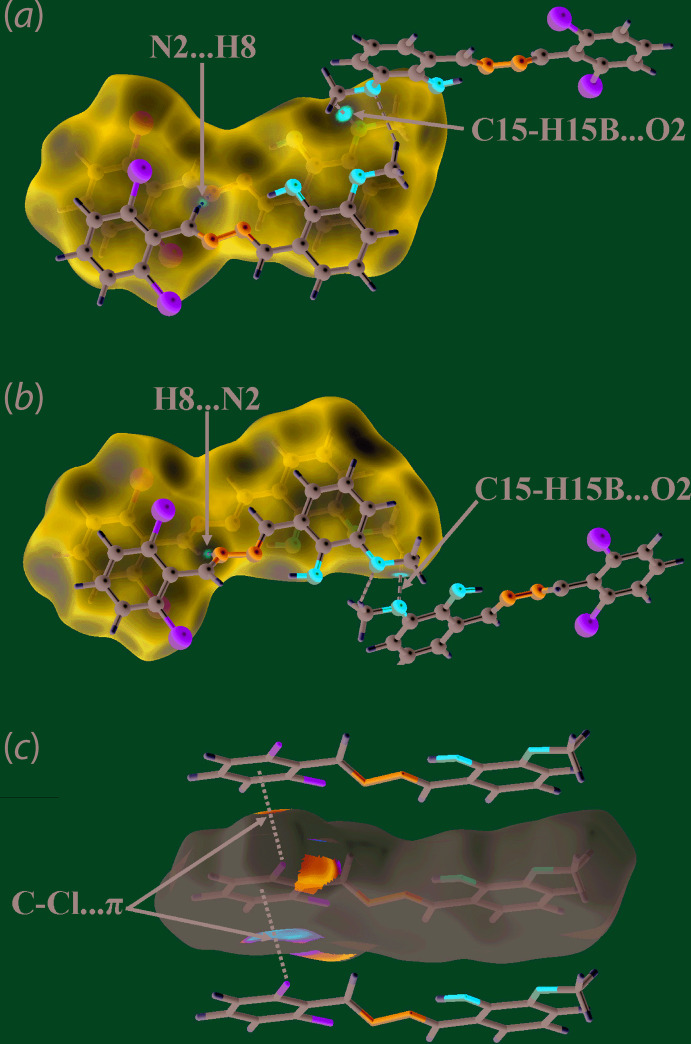
Views of the Hirshfeld surface for (I) mapped: (*a*) and (*b*) over *d*_norm_ in the range −0.097 to + 1.103 arbitrary units and (*c*) with the shape-index property showing inter­molecular C—Cl⋯π/π⋯Cl—C contacts.

**Figure 5:**
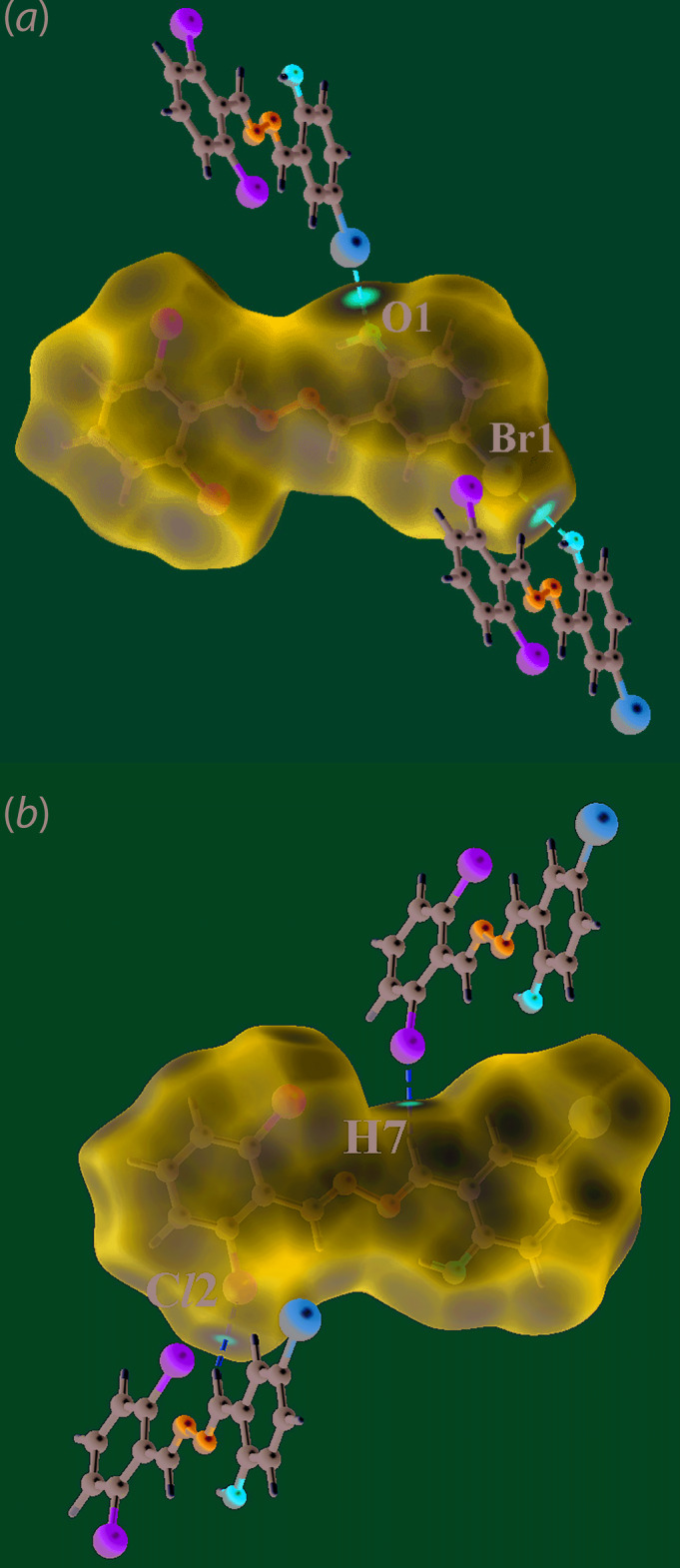
Views of the Hirshfeld surface for (II) mapped over *d*_norm_ in the range −0.016 to 1.528 arbitrary units.

**Figure 6:**
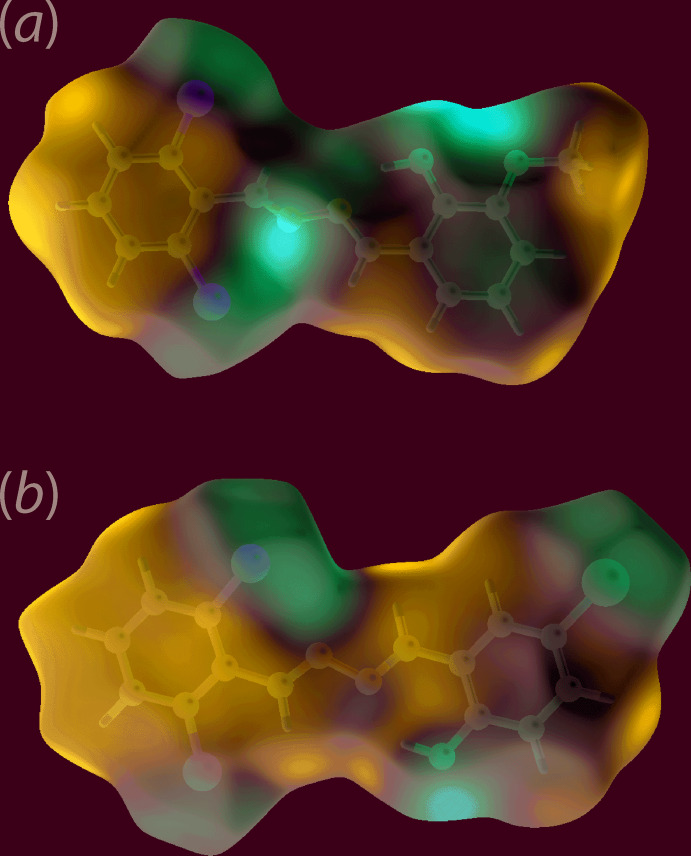
A view of the Hirshfeld surface mapped over the electrostatic potential (the red and blue regions represent negative and positive electrostatic potentials, respectively): (*a*) for (I) in the range −0.071 to +0.038 atomic units and (*b*) for (II) in the range −0.063 to +0.040 atomic units.

On the Hirshfeld surfaces mapped over *d*_norm_, the presence of the bright-red spots near the meth­oxy-O2 and H15*B* atoms for (I) in Fig. 4 ▸(*a*),(*b*) and those near the Br1 and hydroxyl-O1 atoms in Fig. 5 ▸(*a*) for (II), are indicative of dominant inter­molecular C—H⋯O and Br⋯O contacts in their respective crystal structures. The faint-red spots viewed near the imine-N2 and H8 atoms for (I), and near the Cl2 and H7 atoms for (II) in Fig. 4 ▸(*a*),(*b*) and 5(*b*), respectively, indicate the influence of short inter­atomic contacts (Table 4 ▸) on their mol­ecular packing. The Hirshfeld surfaces mapped over the calculated electrostatic potential for (I) and (II) showing contributions from different inter­molecular inter­actions are illustrated through blue and red regions corresponding to positive and negative electrostatic potential in Fig. 6 ▸. For (I), the presence of a short C—Cl2⋯π(C9–C14) contact, Table 2 ▸, is illustrated through a blue bump and a orange concave region in the Hirshfeld surface mapped with the shape-index property in Fig. 4 ▸(*c*).

**Figure 7:**
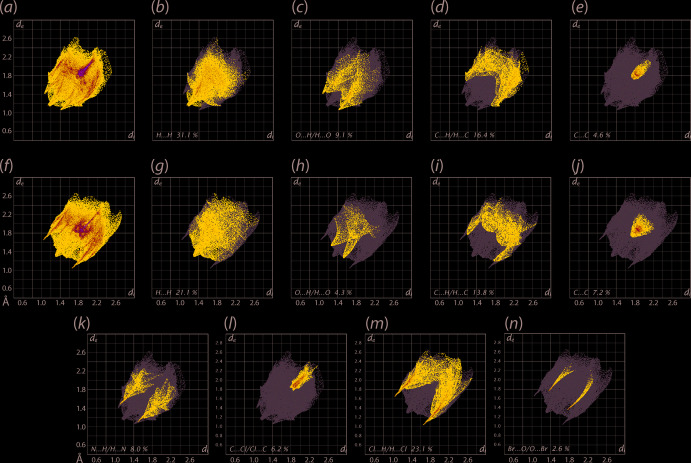
(*a*) A comparison of the full two-dimensional fingerprint plot for (I) and those delineated into (*b*) H⋯H, (*c*) O⋯H/H⋯O, (*d*) C⋯H/H⋯C and (*e*) C⋯C contacts, (*f*)–(*j*) equivalent fingerprint plots for (II), (*g*) N⋯H/H⋯N for (I), (*h*) C⋯Cl/C⋯Cl for (I), (*i*) Cl⋯H/H⋯Cl for (II) and (*j*) Br⋯O/O⋯Br for (II).

The overall two-dimensional fingerprint plots for (I), Fig. 7 ▸(*a*), and (II), Fig.7(*f*), and those delineated into H⋯H, O⋯H/H⋯O, C⋯H/H⋯C and C⋯C contacts for (I) are illustrated in Fig. 7 ▸(*b*)–(*e*), respectively, and the equivalent plots for (II) are found in Fig. 7 ▸(*g*)–(*j*). The percentage contributions from the different inter­atomic contacts to the Hirshfeld surfaces of (I) and (II) are qu­anti­tatively summarized in Table 5 ▸. For (I), the short inter­atomic H⋯H contact between the meth­oxy-H15*A* and di­chloro­benzene-H13 atoms, Table 4 ▸, is evident as a pair of almost fused peaks at *d*_e_ + *d*_i_ ∼2.3 Å in Fig.7(*b*). In (II), comparable inter­actions are at inter­atomic distances farther than the sum of their van der Waals radii. The decrease in the percentage contribution from H⋯H contacts to the Hirshfeld surface of (II) compared to (I), Table 5 ▸, can be related, in the main, to the presence of the bromine substituent in the hydroxyl­benzene ring, in contrast to the meth­oxy group in (I), and its participation in a number of surface contacts, most notably Br⋯H/H⋯Br contacts (13.7%).

The presence of C—H⋯O contacts in the crystal of (I) is characterized as the pair of forceps-like tips at *d*_e_ + *d*_i_ ∼2.5 Å in the fingerprint plot delineated into O⋯H/H⋯O contacts, Fig. 7 ▸(*c*), with the points related to other short inter­atomic O⋯H contacts merged within. The comparatively small contribution from these contacts in (II), Table 5 ▸, show the points to be at distances greater than sum of their van der Waals radii in Fig. 7 ▸(*h*). In the fingerprint plot delineated into C⋯H/H⋯C contacts for both (I) and (II), Fig. 7 ▸(*d*) and (*i*), the characteristic wings are observed but with different shapes. Their relatively long inter­atomic distances are consistent with the absence of inter­molecular C—H⋯π or short C⋯H contacts in the crystals. The absence of aromatic π–π stacking is also evident from the fingerprint plots delineated into C⋯C contacts, Figs. 7(*e*) and (*j*), although significant percentage contributions from these contacts are noted, Table 5 ▸. In addition to the above, some specific contacts occur in the crystals of (I) and (II).

The pair of forceps-like tips at *d*_e_ + *d*_i_ ∼2.5 Å in the fingerprint plot delineated into N⋯H/H⋯N contacts for (I) in Fig. 7 ▸(*k*) indicate the short inter­atomic N⋯H contact involving the imine-N2 and H12 atoms, Table 4 ▸, formed within the supra­molecular chain along *a* axis Fig. 2 ▸(*a*). Also, in the fingerprint plot delineated into C⋯Cl/Cl⋯C contacts for (I), Fig. 7 ▸(*l*), the C—Cl⋯π contacts are highlighted as the pattern of blue points at separations as close as *d*_e_ = *d*_i_ = 1.85 Å. In the case of (II), in the fingerprint plot delineated into Cl⋯H/H⋯Cl contacts, Fig. 7 ▸(*m*), the short inter­atomic contact involving the Cl2 and imine-H7 atoms is apparent as the pair of spikes with their tips at *d*_e_ + *d*_i_ ∼2.7 Å. Finally, the presence of inter­atomic Br⋯O inter­actions along the *a* axis in the crystal is reflected in the pair of thin spikes at *d*_e_ + *d*_i_ ∼3.2 Å in Fig. 7 ▸(*n*). The comparatively greater percentage contribution from inter­atomic contacts such as C⋯O/O⋯C and Cl⋯Cl to the surface of (I) and Br⋯H/H⋯Br and C⋯N/N⋯C to that of (II) as well as smaller contributions from other contacts as summarized in Table 5 ▸, show negligible effect on the respective mol­ecular packing due to the inter­atomic separations being equal to or exceeding the respective sums of the van der Waals radii.

## Energy frameworks   

The pairwise inter­action energies between the mol­ecules in the crystals of (I) and (II) were calculated by summing up four energy components, these being the electrostatic (*E*_ele_), polarization (*E*_pol_), dispersion (*E*_dis_) and exchange-repulsion (*E*_rep_) terms (Turner *et al.*, 2017 ▸). The energies were obtained using the wavefunctions calculated at the B3LYP/6–31 G(*d*,*p*) and HF/STO-3 G levels theory for (I) and (II), respectively. The individual energy components as well as the total inter­action energy were calculated relative to a reference mol­ecule. The nature and strength of the energies for the key identified inter­molecular inter­actions are summarized in Table 6 ▸.

It is apparent from the inter­action energies calculated for (I) that the dispersion component, *E*_dis_, makes the major contribution to the C—Cl⋯π and N⋯H contacts and these are dominant in the mol­ecular packing. By contrast, the C—H⋯O inter­action has nearly equal contributions from the electrostatic component, *E*_ele_, and *E*_dis_. The small value of the inter­action energy corresponding to the short H⋯H contact arises primarily from *E*_dis_. The inter­molecular Br⋯O and Cl⋯H contacts instrumental in the crystal of (II) have small inter­action energy values dominated by *E*_dis_.

**Figure 8:**
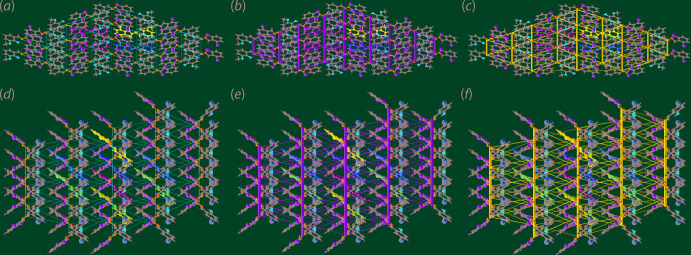
The energy frameworks calculated for (I) showing the (*a*) electrostatic potential force, (*b*) dispersion force and (*c*) total energy. The energy frameworks were adjusted to the same scale factor of 50 with a cut-off value of 3 kJ mol^−1^ within 4 × 4 × 4 unit cells. (*d*)–(*f*) Equivalent frameworks for (II).

Fig. 8 ▸ represents graphically the magnitudes of inter­molecular energies in the form of energy frameworks, which provide a view of the supra­molecular architecture of crystals through cylinders joining centroids of mol­ecular pairs by using red, green and blue colour codes for the components *E*_ele_, *E*_disp_ and *E*_tot_, respectively. The radius of the cylinder is proportional to the magnitude of the inter­action energies which are adjusted to same scale factor of 50 with a cut-off value of 3 kJ mol^−1^ within 4 × 4 × 4 unit cells. The appearance of the energy frameworks clearly reflect the foregoing discussion, namely the clear dominance of the *E*_dis_ terms, especially for (II).

## Database survey   

**Figure 9:**
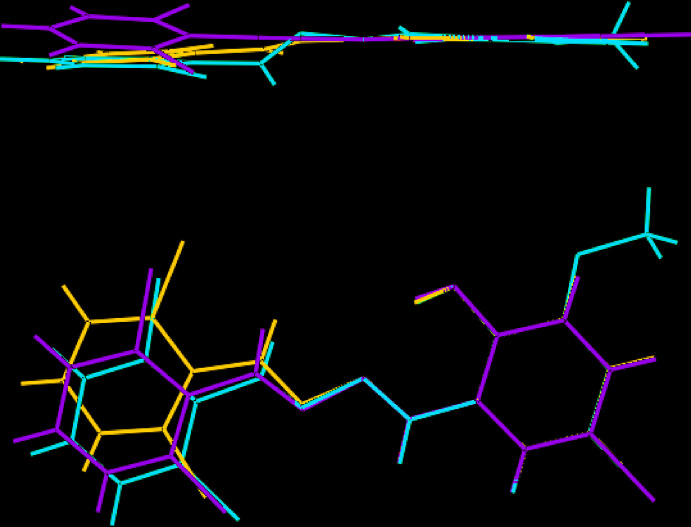
Two overlay diagrams of (I)–(III), represented by red, green and blue images, respectively. The mol­ecules have been overlapped so the O1, N1 and C1 atoms are coincident.

In a recent contribution describing the structure of the analogue of (I) where the meth­oxy substituent is absent (Manawar *et al.*, 2019*b* ▸), *i.e*. (III), it was noted that crystal structure determinations of mol­ecules with the 2-OH-C_6_-C(H)N—NC(H)-C_6_ fragment number fewer than ten, and that there is some conformational flexibility in these mol­ecules. This observation is borne out in the present study where there is a disparity of over 25° in the central C7—N1—N2—C8 torsion angle, *i.e*. −151.0 (3) and 177.8 (6)° for (I) and (II), respectively. These values compare with the equivalent angle of −172.7 (2)° in (III). An overlay diagram for (I)–(III) is shown in Fig. 9 ▸: here, the different conformations for (I), *cf*. (II) and (III), are clearly evident.

## Synthesis and crystallization   

The title compounds were synthesized and characterized as per the procedures reported in the literature (Manawar *et al.*, 2019*a* ▸). The crystals of (I) and (II) in the form of yellow blocks suitable for the structural study reported here were grown by slow evaporation of their chloro­form solutions.

## Refinement   

Crystal data, data collection and structure refinement details are summarized in Table 7 ▸. Carbon-bound H atoms were placed in calculated positions (C—H = 0.93–0.96 Å) and were included in the refinement in the riding-model approximation, with *U*_iso_(H) set to 1.2–1.5*U*_eq_(C). The positions of the O-bound H atoms were refined with O—H = 0.82±0.01 Å, and with *U*_iso_(H) set to 1.5*U*_eq_(O).

## Supplementary Material

Crystal structure: contains datablock(s) . DOI: 10.1107/S2056989020006416/hb7915sup1.cif

Structure factors: contains datablock(s) I. DOI: 10.1107/S2056989020006416/hb7915Isup2.hkl

Structure factors: contains datablock(s) II. DOI: 10.1107/S2056989020006416/hb7915IIsup3.hkl

Supporting information file. DOI: 10.1107/S2056989020006416/hb7915Isup4.cml

Supporting information file. DOI: 10.1107/S2056989020006416/hb7915IIsup5.cml

CCDC references: 2003762, 2003761

Additional supporting information:  crystallographic information; 3D view; checkCIF report

## Figures and Tables

**Table 1:** Selected geometric parameters (Å, °) in (I) and (II)

Parameter	(I)	(II)
N1—N2	1.409 (3)	1.417 (7)
C7—N1	1.283 (3)	1.276 (7)
C8—N2	1.256 (4)	1.234 (7)
N2—N1—C7	112.4 (2)	110.9 (5)
N1—N2—C8	114.2 (2)	114.9 (5)
C1—C7—N1	122.6 (3)	123.1 (6)
C9—C8—N2	121.1 (3)	124.5 (6)
C7—N1—N2—C8	−151.0 (3)	177.8 (6)
C1—C7—N1—N2	−178.8 (2)	−178.9 (5)
C9—C8—N2—N1	179.9 (2)	−179.2 (6)
(C7,N1,N2,C8)/(C1–C6)	20.9 (4)	15.6 (5)
(C7,N1,N2,C8)/(C9–C14)	23.1 (4)	1.5 (6)
(C1–C6)/(C9–C14)	2.41 (17)	15.5 (3)

**Table 2:** Hydrogen-bond geometry (Å, °) for (I) *Cg*1 is the centroid of the (C9–14) ring.

*D*—H⋯*A*	*D*—H	H⋯*A*	*D*⋯*A*	*D*—H⋯*A*
O1—H1*O*⋯N1	0.83 (3)	1.91 (3)	2.657 (3)	149 (3)
C15—H15*B*⋯O2^i^	0.96	2.58	3.439 (4)	149
C14—Cl2⋯*Cg*1^ii^	1.74 (1)	3.70 (1)	3.765 (3)	79 (1)

Symmetry codes: (i) 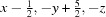; (ii) 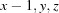.

**Table 3:** Hydrogen-bond geometry (Å, °) for (II)

*D*—H⋯*A*	*D*—H	H⋯*A*	*D*⋯*A*	*D*—H⋯*A*
O1—H1*O*⋯N1	0.83 (6)	1.96 (6)	2.655 (8)	141 (7)

**Table 4:** Summary of short inter­atomic contacts (Å) for (I) and (II)*^*a*^*

Contact	Distance	Symmetry operation
(I)		
H12⋯O1	2.59	−*x*, 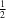 + *y*, 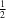 − *z*
H8⋯N2	2.58	−1 + *x*, *y*, *z*
H13⋯H15*A*	2.30	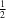 + *x*, 2 − *y*, 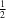 + *z*
(II)		
Br1⋯O1	3.132 (4)	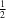 + *x*, 3 − *y*, *z*
Cl2⋯H7	2.69	− 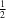 + *x*, 1 − *y*, *z*

Notes: (*a*) The inter­atomic distances are calculated in *Crystal Explorer* (Turner *et al.*, 2017 ▸) whereby the *X*—H bond lengths are adjusted to their neutron values.

**Table 5:** Percentage contributions of inter­atomic contacts to the Hirshfeld surface for (I) and (II)

Contact		Percentage contribution
	(I)	(II)
H⋯H	31.1	21.1
O⋯H/H⋯O	9.1	4.3
C⋯H/H⋯C	16.4	13.8
Cl⋯H/H⋯Cl	17.3	23.1
N⋯H/H⋯N	8.0	0.4
C⋯Cl/Cl⋯C	6.2	1.0
C⋯C	4.6	7.2
C⋯O/O⋯C	3.7	0.1
C⋯N/N⋯C	0.0	7.1
Cl⋯Cl	3.5	2.7
Cl⋯N/N⋯Cl	0.0	0.6
N⋯O/O⋯N	0.0	0.1
Br⋯H/H⋯Br	–	13.7
Br⋯O/O⋯Br	–	2.6
Br⋯C/C⋯Br	–	1.8
Br⋯Cl/Cl⋯Br	–	0.2
Br⋯Br	–	0.2

**Table 6:** Summary of inter­action energies (kJ mol^−1^) calculated for (I) and (II)

Contact	*R* (Å)	*E*_ele_	*E*_pol_	*E*_dis_	*E*_rep_	*E*_tot_
(I)*^*a*^*						
C15—H15*B*⋯O2^i^	12.93	−12.5	−2.7	−13.1	9.1	−21.1
C14—Cl2 ⋯π(C9–C14)^ii^ +	4.36	−4.7	−3.5	−66.8	36.9	−43.0
N2 ⋯H8^iii^						
H13⋯H15*A*′^iv^	13.64	−0.6	−0.6	−9.7	5.5	−6.1
(II)*^*b*^*						
Br1⋯O1^i^	10.21	−4.6	−0.9	−7.2	5.4	−7.5
Cl2⋯H7^ii^	8.69	−3.9	−0.7	−4.2	0.7	−3.1

Notes: (*a*) Symmetry operations for (I): (i) −1 + *x*, *y*, *z*; (ii) −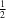 + *x*, 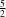 − *y*, − *z*; (iii) 1 + *x*, *y*, *z*. (*b*) Symmetry operations for (II): (i) 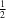 + *x*, 3 − *y*, *z*; (ii) −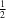 + *x*, 1 − *y*, *z*.

**Table 7:** Experimental details

	(I)	(II)
Crystal data		
Chemical formula	C_15_H_12_Cl_2_N_2_O_2_	C_14_H_9_BrCl_2_N_2_O
*M*_r_	323.17	372.04
Crystal system, space group	Orthorhombic, *P*2_1_2_1_2_1_	Orthorhombic, *P* *c* *a*2_1_
Temperature (K)	296	296
*a*, *b*, *c* (Å)	4.3556 (2), 12.8548 (4), 25.9904 (9)	16.4510 (12), 4.4314 (3), 20.0523 (15)
*V* (Å^3^)	1455.21 (10)	1461.83 (18)
*Z*	4	4
Radiation type	Mo *K*α	Mo *K*α
μ (mm^−1^)	0.45	3.17
Crystal size (mm)	0.30 × 0.25 × 0.25	0.30 × 0.20 × 0.20

Data collection		
Diffractometer	Bruker Kappa APEXII CCD	Bruker Kappa APEXII CCD
Absorption correction	Multi-scan (*SADABS*; Bruker, 2004 ▸)	Multi-scan (*SADABS*; Bruker, 2004 ▸)
*T*_min_, *T*_max_	0.557, 0.746	0.398, 0.746
No. of measured, independent and observed [*I* > 2σ(*I*)] reflections	48935, 3751, 2909	39831, 3569, 2150
*R*_int_	0.070	0.108
(sin θ/λ)_max_ (Å^−1^)	0.678	0.666

Refinement		
*R*[*F* ^2^ > 2σ(*F* ^2^)], *wR*(*F* ^2^), *S*	0.039, 0.099, 1.02	0.038, 0.082, 1.00
No. of reflections	3751	3569
No. of parameters	194	184
No. of restraints	1	2
H-atom treatment	H atoms treated by a mixture of independent and constrained refinement	H atoms treated by a mixture of independent and constrained refinement
Δρ_max_, Δρ_min_ (e Å^−3^)	0.16, −0.25	0.30, −0.59
Absolute structure	Flack *x* determined using 1004 quotients [(*I* ^+^)−(*I* ^−^)]/[(*I* ^+^)+(*I* ^−^)] (Parsons *et al.*, 2013 ▸).	Flack *x* determined using 829 quotients [(*I* ^+^)−(*I* ^−^)]/[(*I* ^+^)+(*I* ^−^)] (Parsons *et al.*, 2013 ▸).
Absolute structure parameter	0.12 (3)	0.003 (7)

Computer programs: *APEX2* and *SAINT* (Bruker, 2004 ▸), *SIR92* (Altomare *et al.*, 1994 ▸), *SHELXL2014/7* (Sheldrick, 2015 ▸), *ORTEP-3 for Windows* (Farrugia, 2012 ▸), *DIAMOND* (Brandenburg, 2006 ▸) and *publCIF* (Westrip, 2010 ▸).

## supplementary crystallographic information

### 2-({(E)-2-[(E)-2,6-Dichlorobenzylidene]hydrazin-1-ylidene}methyl)-6-methoxyphenol (I) Crystal data

**Table:**

C_15_H_12_Cl_2_N_2_O_2_	*D*_x_ = 1.475 Mg m^−^^3^
*M**_r_* = 323.17	Mo *K*α radiation, λ = 0.71073 Å
Orthorhombic, *P*2_1_2_1_2_1_	Cell parameters from 7669 reflections
*a* = 4.3556 (2) Å	θ = 2.8–20.9°
*b* = 12.8548 (4) Å	µ = 0.45 mm^−^^1^
*c* = 25.9904 (9) Å	*T* = 296 K
*V* = 1455.21 (10) Å^3^	Block, yellow
*Z* = 4	0.30 × 0.25 × 0.25 mm
*F*(000) = 664	

### 2-({(E)-2-[(E)-2,6-Dichlorobenzylidene]hydrazin-1-ylidene}methyl)-6-methoxyphenol (I) Data collection

**Table:**

Bruker Kappa APEXII CCD diffractometer	2909 reflections with *I* > 2σ(*I*)
Radiation source: X-ray tube	*R*_int_ = 0.070
ω and φ scan	θ_max_ = 28.8°, θ_min_ = 1.6°
Absorption correction: multi-scan (SADABS; Bruker, 2004)	*h* = −5→5
*T*_min_ = 0.557, *T*_max_ = 0.746	*k* = −17→17
48935 measured reflections	*l* = −34→35
3751 independent reflections	

### 2-({(E)-2-[(E)-2,6-Dichlorobenzylidene]hydrazin-1-ylidene}methyl)-6-methoxyphenol (I) Refinement

**Table:**

Refinement on *F*^2^	Secondary atom site location: difference Fourier map
Least-squares matrix: full	Hydrogen site location: mixed
*R*[*F*^2^ > 2σ(*F*^2^)] = 0.039	H atoms treated by a mixture of independent and constrained refinement
*wR*(*F*^2^) = 0.099	*w* = 1/[σ^2^(*F*_o_^2^) + (0.0465*P*)^2^ + 0.2295*P*] where *P* = (*F*_o_^2^ + 2*F*_c_^2^)/3
*S* = 1.02	(Δ/σ)_max_ < 0.001
3751 reflections	Δρ_max_ = 0.16 e Å^−^^3^
194 parameters	Δρ_min_ = −0.25 e Å^−^^3^
1 restraint	Absolute structure: Flack *x* determined using 1004 quotients [(*I*^+^)-(*I*^-^)]/[(*I*^+^)+(*I*^-^)] (Parsons *et al.*, 2013).
Primary atom site location: structure-invariant direct methods	Absolute structure parameter: 0.12 (3)

### 2-({(E)-2-[(E)-2,6-Dichlorobenzylidene]hydrazin-1-ylidene}methyl)-6-methoxyphenol (I) Special details

**Table:**

Geometry. All esds (except the esd in the dihedral angle between two l.s. planes) are estimated using the full covariance matrix. The cell esds are taken into account individually in the estimation of esds in distances, angles and torsion angles; correlations between esds in cell parameters are only used when they are defined by crystal symmetry. An approximate (isotropic) treatment of cell esds is used for estimating esds involving l.s. planes.

### 2-({(E)-2-[(E)-2,6-Dichlorobenzylidene]hydrazin-1-ylidene}methyl)-6-methoxyphenol (I) Fractional atomic coordinates and isotropic or equivalent isotropic displacement parameters (Å^2^)

**Table:**

	*x*	*y*	*z*	*U*_iso_*/*U*_eq_	
Cl1	0.5635 (2)	0.58257 (6)	0.17813 (3)	0.0639 (2)	
Cl2	−0.1091 (2)	0.85695 (7)	0.29187 (3)	0.0635 (2)	
O1	0.0486 (6)	1.03357 (16)	0.07165 (7)	0.0526 (5)	
H1O	0.076 (10)	0.990 (2)	0.0947 (10)	0.079*	
O2	−0.0282 (6)	1.13830 (16)	−0.01337 (7)	0.0580 (6)	
N1	0.2888 (6)	0.86954 (18)	0.11908 (9)	0.0462 (6)	
N2	0.3817 (6)	0.80405 (19)	0.15969 (9)	0.0511 (6)	
C1	0.3847 (6)	0.9094 (2)	0.03056 (9)	0.0407 (6)	
C2	0.1975 (6)	0.9977 (2)	0.02967 (9)	0.0406 (6)	
C3	0.1588 (7)	1.0536 (2)	−0.01676 (10)	0.0436 (6)	
C4	0.3053 (7)	1.0193 (2)	−0.06085 (10)	0.0529 (8)	
H4	0.280990	1.055923	−0.091441	0.063*	
C5	0.4876 (8)	0.9312 (3)	−0.05996 (11)	0.0577 (8)	
H5	0.581625	0.908278	−0.090022	0.069*	
C6	0.5300 (8)	0.8777 (2)	−0.01501 (11)	0.0531 (7)	
H6	0.656910	0.819478	−0.014652	0.064*	
C7	0.4354 (7)	0.8510 (2)	0.07726 (10)	0.0454 (6)	
H7	0.580111	0.797798	0.077020	0.054*	
C8	0.1755 (7)	0.7862 (2)	0.19229 (10)	0.0433 (6)	
H8	−0.017552	0.815862	0.188063	0.052*	
C9	0.2348 (6)	0.7189 (2)	0.23722 (10)	0.0384 (6)	
C10	0.4098 (7)	0.6277 (2)	0.23543 (10)	0.0442 (6)	
C11	0.4597 (8)	0.5674 (2)	0.27870 (12)	0.0559 (8)	
H11	0.578168	0.507359	0.276454	0.067*	
C12	0.3337 (8)	0.5965 (3)	0.32511 (12)	0.0597 (8)	
H12	0.370349	0.556547	0.354312	0.072*	
C13	0.1540 (8)	0.6841 (3)	0.32858 (11)	0.0543 (8)	
H13	0.064794	0.702856	0.359734	0.065*	
C14	0.1081 (7)	0.7439 (2)	0.28499 (10)	0.0439 (6)	
C15	−0.0503 (10)	1.2040 (3)	−0.05738 (12)	0.0743 (11)	
H15A	0.146096	1.235029	−0.064168	0.111*	
H15B	−0.198696	1.257741	−0.051006	0.111*	
H15C	−0.113212	1.163644	−0.086601	0.111*	

### 2-({(E)-2-[(E)-2,6-Dichlorobenzylidene]hydrazin-1-ylidene}methyl)-6-methoxyphenol (I) Atomic displacement parameters (Å^2^)

**Table:**

	*U*^11^	*U*^22^	*U*^33^	*U*^12^	*U*^13^	*U*^23^
Cl1	0.0822 (6)	0.0538 (4)	0.0555 (4)	0.0136 (4)	0.0063 (4)	−0.0055 (4)
Cl2	0.0727 (5)	0.0550 (4)	0.0628 (5)	0.0050 (4)	0.0086 (4)	−0.0072 (4)
O1	0.0699 (14)	0.0525 (12)	0.0353 (10)	0.0110 (11)	0.0077 (10)	0.0009 (8)
O2	0.0781 (15)	0.0535 (12)	0.0425 (11)	0.0107 (12)	0.0016 (10)	0.0082 (9)
N1	0.0520 (14)	0.0456 (13)	0.0412 (13)	−0.0021 (11)	−0.0001 (11)	0.0106 (10)
N2	0.0489 (13)	0.0557 (14)	0.0486 (13)	0.0001 (12)	−0.0027 (12)	0.0156 (11)
C1	0.0438 (14)	0.0426 (14)	0.0356 (13)	−0.0066 (12)	0.0008 (11)	−0.0029 (11)
C2	0.0476 (15)	0.0438 (14)	0.0303 (12)	−0.0066 (12)	0.0019 (11)	−0.0054 (11)
C3	0.0493 (15)	0.0448 (15)	0.0366 (13)	−0.0063 (13)	−0.0020 (12)	−0.0002 (11)
C4	0.0604 (19)	0.066 (2)	0.0325 (14)	−0.0094 (16)	0.0021 (13)	0.0015 (14)
C5	0.064 (2)	0.0714 (19)	0.0377 (15)	−0.0019 (18)	0.0109 (14)	−0.0091 (14)
C6	0.0553 (17)	0.0555 (17)	0.0484 (16)	−0.0004 (15)	0.0075 (14)	−0.0117 (13)
C7	0.0469 (16)	0.0408 (14)	0.0485 (15)	−0.0024 (13)	−0.0020 (13)	0.0001 (12)
C8	0.0494 (16)	0.0414 (14)	0.0390 (14)	−0.0027 (13)	−0.0062 (12)	0.0021 (11)
C9	0.0406 (13)	0.0377 (13)	0.0370 (13)	−0.0076 (11)	−0.0051 (11)	0.0022 (10)
C10	0.0472 (15)	0.0433 (14)	0.0420 (14)	−0.0030 (13)	−0.0037 (13)	0.0007 (12)
C11	0.0602 (18)	0.0476 (16)	0.0600 (18)	0.0029 (15)	−0.0070 (15)	0.0143 (14)
C12	0.066 (2)	0.065 (2)	0.0481 (17)	−0.0039 (17)	−0.0051 (16)	0.0233 (15)
C13	0.0587 (18)	0.069 (2)	0.0355 (14)	−0.0093 (16)	−0.0006 (13)	0.0059 (14)
C14	0.0456 (15)	0.0442 (14)	0.0419 (14)	−0.0082 (12)	−0.0026 (13)	−0.0016 (12)
C15	0.098 (3)	0.072 (2)	0.0529 (19)	0.014 (2)	−0.001 (2)	0.0195 (17)

### 2-({(E)-2-[(E)-2,6-Dichlorobenzylidene]hydrazin-1-ylidene}methyl)-6-methoxyphenol (I) Geometric parameters (Å, º)

**Table:**

Cl1—C10	1.733 (3)	C5—H5	0.9300
Cl2—C14	1.743 (3)	C6—H6	0.9300
O1—C2	1.350 (3)	C7—H7	0.9300
O1—H1O	0.828 (13)	C8—C9	1.476 (4)
O2—C3	1.362 (4)	C8—H8	0.9300
O2—C15	1.425 (3)	C9—C10	1.399 (4)
N1—C7	1.283 (3)	C9—C14	1.396 (4)
N1—N2	1.409 (3)	C10—C11	1.383 (4)
N2—C8	1.256 (4)	C11—C12	1.377 (4)
C1—C2	1.398 (4)	C11—H11	0.9300
C1—C6	1.403 (4)	C12—C13	1.374 (5)
C1—C7	1.444 (4)	C12—H12	0.9300
C2—C3	1.415 (4)	C13—C14	1.384 (4)
C3—C4	1.384 (4)	C13—H13	0.9300
C4—C5	1.383 (4)	C15—H15A	0.9600
C4—H4	0.9300	C15—H15B	0.9600
C5—C6	1.369 (4)	C15—H15C	0.9600
			
C2—O1—H1O	107 (3)	N2—C8—H8	119.5
C3—O2—C15	117.5 (3)	C9—C8—H8	119.5
C7—N1—N2	112.4 (2)	C10—C9—C14	116.0 (2)
C8—N2—N1	114.2 (2)	C10—C9—C8	124.1 (2)
C2—C1—C6	119.0 (3)	C14—C9—C8	120.0 (3)
C2—C1—C7	121.7 (2)	C11—C10—C9	121.9 (3)
C6—C1—C7	119.3 (3)	C11—C10—Cl1	116.8 (2)
O1—C2—C1	123.0 (2)	C9—C10—Cl1	121.3 (2)
O1—C2—C3	117.3 (3)	C10—C11—C12	119.8 (3)
C1—C2—C3	119.8 (2)	C10—C11—H11	120.1
O2—C3—C4	125.7 (3)	C12—C11—H11	120.1
O2—C3—C2	115.0 (2)	C13—C12—C11	120.5 (3)
C4—C3—C2	119.3 (3)	C13—C12—H12	119.8
C3—C4—C5	120.8 (3)	C11—C12—H12	119.8
C3—C4—H4	119.6	C12—C13—C14	118.9 (3)
C5—C4—H4	119.6	C12—C13—H13	120.5
C6—C5—C4	120.2 (3)	C14—C13—H13	120.5
C6—C5—H5	119.9	C13—C14—C9	122.9 (3)
C4—C5—H5	119.9	C13—C14—Cl2	117.3 (2)
C5—C6—C1	120.9 (3)	C9—C14—Cl2	119.8 (2)
C5—C6—H6	119.5	O2—C15—H15A	109.5
C1—C6—H6	119.5	O2—C15—H15B	109.5
N1—C7—C1	122.6 (3)	H15A—C15—H15B	109.5
N1—C7—H7	118.7	O2—C15—H15C	109.5
C1—C7—H7	118.7	H15A—C15—H15C	109.5
N2—C8—C9	121.1 (3)	H15B—C15—H15C	109.5
			
C7—N1—N2—C8	−151.0 (3)	C6—C1—C7—N1	−173.4 (3)
C6—C1—C2—O1	179.9 (3)	N1—N2—C8—C9	179.9 (2)
C7—C1—C2—O1	−1.0 (4)	N2—C8—C9—C10	−39.5 (4)
C6—C1—C2—C3	−0.3 (4)	N2—C8—C9—C14	141.8 (3)
C7—C1—C2—C3	178.8 (3)	C14—C9—C10—C11	−1.4 (4)
C15—O2—C3—C4	−7.2 (5)	C8—C9—C10—C11	179.7 (3)
C15—O2—C3—C2	173.5 (3)	C14—C9—C10—Cl1	176.3 (2)
O1—C2—C3—O2	−0.2 (4)	C8—C9—C10—Cl1	−2.5 (4)
C1—C2—C3—O2	180.0 (2)	C9—C10—C11—C12	0.4 (5)
O1—C2—C3—C4	−179.6 (3)	Cl1—C10—C11—C12	−177.4 (2)
C1—C2—C3—C4	0.6 (4)	C10—C11—C12—C13	1.1 (5)
O2—C3—C4—C5	−179.2 (3)	C11—C12—C13—C14	−1.5 (5)
C2—C3—C4—C5	0.1 (4)	C12—C13—C14—C9	0.5 (4)
C3—C4—C5—C6	−1.2 (5)	C12—C13—C14—Cl2	−177.6 (3)
C4—C5—C6—C1	1.5 (5)	C10—C9—C14—C13	1.0 (4)
C2—C1—C6—C5	−0.8 (4)	C8—C9—C14—C13	179.9 (3)
C7—C1—C6—C5	−179.9 (3)	C10—C9—C14—Cl2	179.0 (2)
N2—N1—C7—C1	−178.8 (2)	C8—C9—C14—Cl2	−2.1 (4)
C2—C1—C7—N1	7.5 (4)		

### 2-({(E)-2-[(E)-2,6-Dichlorobenzylidene]hydrazin-1-ylidene}methyl)-6-methoxyphenol (I) Hydrogen-bond geometry (Å, º)

*Cg*1 is the centroid of the (C9–14) ring.

**Table:**

*D*—H···*A*	*D*—H	H···*A*	*D*···*A*	*D*—H···*A*
O1—H1*O*···N1	0.83 (3)	1.91 (3)	2.657 (3)	149 (3)
C15—H15*B*···O2^i^	0.96	2.58	3.439 (4)	149
C14—Cl2···*Cg*1^ii^	1.74 (1)	3.70 (1)	3.765 (3)	79 (1)

Symmetry codes: (i) *x*−1/2, −*y*+5/2, −*z*; (ii) *x*−1, *y*, *z*.

### 4-Bromo-2-({(E)-2-[(E)-2,6-dichlorobenzylidene]hydrazin-1-ylidene}methyl)phenol (II) Crystal data

**Table:**

C_14_H_9_BrCl_2_N_2_O	*D*_x_ = 1.690 Mg m^−^^3^
*M**_r_* = 372.04	Mo *K*α radiation, λ = 0.71073 Å
Orthorhombic, *P**c**a*2_1_	Cell parameters from 4698 reflections
*a* = 16.4510 (12) Å	θ = 2.5–18.6°
*b* = 4.4314 (3) Å	µ = 3.17 mm^−^^1^
*c* = 20.0523 (15) Å	*T* = 296 K
*V* = 1461.83 (18) Å^3^	Block, yellow
*Z* = 4	0.30 × 0.20 × 0.20 mm
*F*(000) = 736	

### 4-Bromo-2-({(E)-2-[(E)-2,6-dichlorobenzylidene]hydrazin-1-ylidene}methyl)phenol (II) Data collection

**Table:**

Bruker Kappa APEXII CCD diffractometer	2150 reflections with *I* > 2σ(*I*)
Radiation source: X-ray tube	*R*_int_ = 0.108
ω and φ scan	θ_max_ = 28.3°, θ_min_ = 2.5°
Absorption correction: multi-scan (SADABS; Bruker, 2004)	*h* = −21→21
*T*_min_ = 0.398, *T*_max_ = 0.746	*k* = −5→5
39831 measured reflections	*l* = −26→26
3569 independent reflections	

### 4-Bromo-2-({(E)-2-[(E)-2,6-dichlorobenzylidene]hydrazin-1-ylidene}methyl)phenol (II) Refinement

**Table:**

Refinement on *F*^2^	Secondary atom site location: difference Fourier map
Least-squares matrix: full	Hydrogen site location: mixed
*R*[*F*^2^ > 2σ(*F*^2^)] = 0.038	H atoms treated by a mixture of independent and constrained refinement
*wR*(*F*^2^) = 0.082	*w* = 1/[σ^2^(*F*_o_^2^) + (0.024*P*)^2^ + 0.3263*P*] where *P* = (*F*_o_^2^ + 2*F*_c_^2^)/3
*S* = 1.00	(Δ/σ)_max_ < 0.001
3569 reflections	Δρ_max_ = 0.30 e Å^−^^3^
184 parameters	Δρ_min_ = −0.59 e Å^−^^3^
2 restraints	Absolute structure: Flack *x* determined using 829 quotients [(*I*^+^)-(*I*^-^)]/[(*I*^+^)+(*I*^-^)] (Parsons *et al.*, 2013).
Primary atom site location: structure-invariant direct methods	Absolute structure parameter: 0.003 (7)

### 4-Bromo-2-({(E)-2-[(E)-2,6-dichlorobenzylidene]hydrazin-1-ylidene}methyl)phenol (II) Special details

**Table:**

Geometry. All esds (except the esd in the dihedral angle between two l.s. planes) are estimated using the full covariance matrix. The cell esds are taken into account individually in the estimation of esds in distances, angles and torsion angles; correlations between esds in cell parameters are only used when they are defined by crystal symmetry. An approximate (isotropic) treatment of cell esds is used for estimating esds involving l.s. planes.

### 4-Bromo-2-({(E)-2-[(E)-2,6-dichlorobenzylidene]hydrazin-1-ylidene}methyl)phenol (II) Fractional atomic coordinates and isotropic or equivalent isotropic displacement parameters (Å^2^)

**Table:**

	*x*	*y*	*z*	*U*_iso_*/*U*_eq_	
Br1	1.17606 (4)	1.58575 (14)	0.09985 (4)	0.0617 (2)	
Cl1	0.95249 (10)	0.2117 (4)	0.37299 (9)	0.0682 (5)	
Cl2	0.63270 (11)	0.3504 (5)	0.30362 (11)	0.0830 (6)	
O1	0.8392 (2)	1.0502 (9)	0.1011 (4)	0.0618 (10)	
H1O	0.830 (5)	0.931 (14)	0.132 (3)	0.093*	
N1	0.8804 (3)	0.7671 (11)	0.2128 (2)	0.0459 (12)	
N2	0.8764 (3)	0.5871 (12)	0.2711 (3)	0.0583 (14)	
C1	0.9706 (3)	1.0963 (12)	0.1535 (3)	0.0395 (13)	
C2	0.9148 (3)	1.1674 (11)	0.1024 (4)	0.0461 (13)	
C3	0.9379 (4)	1.3636 (14)	0.0523 (3)	0.0568 (18)	
H3	0.901334	1.412779	0.018641	0.068*	
C4	1.0152 (4)	1.4872 (14)	0.0519 (3)	0.0557 (17)	
H4	1.029972	1.620146	0.018149	0.067*	
C5	1.0700 (3)	1.4155 (11)	0.1008 (5)	0.0454 (12)	
C6	1.0480 (4)	1.2229 (13)	0.1514 (3)	0.0450 (15)	
H6	1.085312	1.176591	0.184695	0.054*	
C7	0.9496 (4)	0.8944 (13)	0.2077 (3)	0.0458 (14)	
H7	0.988542	0.855756	0.240209	0.055*	
C8	0.8102 (4)	0.4636 (14)	0.2809 (3)	0.0490 (15)	
H8	0.769260	0.494996	0.249745	0.059*	
C9	0.7918 (4)	0.2707 (15)	0.3384 (3)	0.0441 (15)	
C10	0.8495 (4)	0.1490 (13)	0.3823 (3)	0.0471 (15)	
C11	0.8267 (4)	−0.0303 (14)	0.4358 (3)	0.0602 (17)	
H11	0.866252	−0.109704	0.463911	0.072*	
C12	0.7460 (5)	−0.0916 (16)	0.4478 (4)	0.073 (2)	
H12	0.731322	−0.211614	0.483867	0.087*	
C13	0.6868 (4)	0.0252 (16)	0.4062 (4)	0.066 (2)	
H13	0.632125	−0.015359	0.413876	0.079*	
C14	0.7104 (4)	0.2044 (17)	0.3525 (3)	0.0525 (18)	

### 4-Bromo-2-({(E)-2-[(E)-2,6-dichlorobenzylidene]hydrazin-1-ylidene}methyl)phenol (II) Atomic displacement parameters (Å^2^)

**Table:**

	*U*^11^	*U*^22^	*U*^33^	*U*^12^	*U*^13^	*U*^23^
Br1	0.0534 (4)	0.0635 (3)	0.0682 (4)	−0.0060 (3)	0.0127 (4)	−0.0003 (5)
Cl1	0.0462 (9)	0.0986 (13)	0.0599 (10)	0.0036 (9)	−0.0082 (8)	0.0084 (10)
Cl2	0.0440 (10)	0.1108 (15)	0.0943 (14)	−0.0090 (11)	−0.0136 (10)	0.0087 (13)
O1	0.048 (2)	0.068 (3)	0.069 (3)	−0.003 (2)	−0.015 (3)	0.010 (3)
N1	0.047 (3)	0.044 (3)	0.047 (3)	0.000 (3)	−0.004 (2)	−0.001 (2)
N2	0.048 (4)	0.067 (4)	0.060 (4)	−0.011 (3)	−0.011 (3)	0.019 (3)
C1	0.043 (3)	0.036 (3)	0.039 (3)	0.003 (3)	0.001 (3)	−0.004 (3)
C2	0.046 (3)	0.044 (3)	0.048 (3)	0.005 (2)	−0.005 (4)	−0.001 (4)
C3	0.063 (5)	0.054 (4)	0.053 (4)	0.009 (3)	−0.013 (3)	0.005 (3)
C4	0.068 (5)	0.049 (4)	0.050 (4)	0.001 (3)	0.004 (4)	0.009 (3)
C5	0.053 (3)	0.038 (3)	0.045 (3)	0.003 (3)	0.006 (4)	−0.003 (4)
C6	0.043 (4)	0.046 (3)	0.046 (4)	0.004 (3)	−0.001 (3)	−0.002 (3)
C7	0.042 (4)	0.049 (3)	0.047 (3)	0.004 (3)	−0.005 (3)	0.002 (3)
C8	0.041 (4)	0.059 (4)	0.047 (4)	0.002 (3)	−0.007 (3)	0.000 (3)
C9	0.044 (4)	0.046 (4)	0.042 (4)	−0.008 (3)	−0.001 (3)	−0.006 (3)
C10	0.048 (4)	0.050 (4)	0.044 (3)	−0.004 (3)	0.001 (3)	−0.008 (3)
C11	0.072 (5)	0.063 (4)	0.046 (4)	−0.004 (4)	−0.005 (4)	−0.002 (3)
C12	0.081 (6)	0.078 (5)	0.058 (4)	−0.021 (5)	0.014 (5)	−0.003 (4)
C13	0.054 (4)	0.080 (5)	0.063 (4)	−0.023 (4)	0.012 (4)	−0.010 (4)
C14	0.046 (4)	0.065 (4)	0.047 (4)	−0.011 (4)	0.005 (3)	−0.007 (3)

### 4-Bromo-2-({(E)-2-[(E)-2,6-dichlorobenzylidene]hydrazin-1-ylidene}methyl)phenol (II) Geometric parameters (Å, º)

**Table:**

Br1—C5	1.901 (5)	C4—H4	0.9300
Cl1—C10	1.727 (6)	C5—C6	1.375 (10)
Cl2—C14	1.736 (8)	C6—H6	0.9300
O1—C2	1.349 (7)	C7—H7	0.9300
O1—H1O	0.827 (14)	C8—C9	1.466 (9)
N1—C7	1.276 (7)	C8—H8	0.9300
N1—N2	1.417 (7)	C9—C10	1.402 (9)
N2—C8	1.234 (7)	C9—C14	1.400 (8)
C1—C6	1.393 (8)	C10—C11	1.388 (9)
C1—C2	1.411 (9)	C11—C12	1.375 (10)
C1—C7	1.448 (8)	C11—H11	0.9300
C2—C3	1.382 (9)	C12—C13	1.384 (11)
C3—C4	1.384 (9)	C12—H12	0.9300
C3—H3	0.9300	C13—C14	1.392 (10)
C4—C5	1.369 (10)	C13—H13	0.9300
			
C2—O1—H1O	114 (6)	C1—C7—H7	118.5
C7—N1—N2	110.9 (5)	N2—C8—C9	124.5 (6)
C8—N2—N1	114.9 (5)	N2—C8—H8	117.8
C6—C1—C2	118.8 (5)	C9—C8—H8	117.8
C6—C1—C7	119.3 (5)	C10—C9—C14	116.1 (6)
C2—C1—C7	121.9 (5)	C10—C9—C8	125.3 (6)
O1—C2—C3	118.8 (6)	C14—C9—C8	118.6 (6)
O1—C2—C1	121.9 (6)	C11—C10—C9	121.5 (6)
C3—C2—C1	119.3 (5)	C11—C10—Cl1	116.2 (5)
C2—C3—C4	120.4 (6)	C9—C10—Cl1	122.3 (5)
C2—C3—H3	119.8	C12—C11—C10	120.6 (7)
C4—C3—H3	119.8	C12—C11—H11	119.7
C5—C4—C3	120.6 (6)	C10—C11—H11	119.7
C5—C4—H4	119.7	C11—C12—C13	120.0 (7)
C3—C4—H4	119.7	C11—C12—H12	120.0
C4—C5—C6	119.9 (5)	C13—C12—H12	120.0
C4—C5—Br1	120.4 (6)	C12—C13—C14	118.9 (7)
C6—C5—Br1	119.7 (6)	C12—C13—H13	120.6
C5—C6—C1	120.9 (6)	C14—C13—H13	120.6
C5—C6—H6	119.5	C13—C14—C9	122.9 (6)
C1—C6—H6	119.5	C13—C14—Cl2	116.3 (6)
N1—C7—C1	123.1 (6)	C9—C14—Cl2	120.8 (5)
N1—C7—H7	118.5		
			
C7—N1—N2—C8	177.8 (6)	N1—N2—C8—C9	−179.2 (6)
C6—C1—C2—O1	179.3 (6)	N2—C8—C9—C10	−13.9 (10)
C7—C1—C2—O1	−0.2 (9)	N2—C8—C9—C14	164.6 (7)
C6—C1—C2—C3	−1.0 (8)	C14—C9—C10—C11	1.0 (9)
C7—C1—C2—C3	179.4 (5)	C8—C9—C10—C11	179.5 (6)
O1—C2—C3—C4	−179.7 (6)	C14—C9—C10—Cl1	−179.2 (5)
C1—C2—C3—C4	0.6 (9)	C8—C9—C10—Cl1	−0.6 (9)
C2—C3—C4—C5	0.5 (10)	C9—C10—C11—C12	−0.6 (10)
C3—C4—C5—C6	−1.1 (10)	Cl1—C10—C11—C12	179.5 (5)
C3—C4—C5—Br1	179.9 (5)	C10—C11—C12—C13	0.1 (10)
C4—C5—C6—C1	0.6 (9)	C11—C12—C13—C14	−0.1 (11)
Br1—C5—C6—C1	179.6 (4)	C12—C13—C14—C9	0.5 (11)
C2—C1—C6—C5	0.4 (8)	C12—C13—C14—Cl2	−178.5 (6)
C7—C1—C6—C5	180.0 (5)	C10—C9—C14—C13	−0.9 (10)
N2—N1—C7—C1	−178.9 (5)	C8—C9—C14—C13	−179.6 (6)
C6—C1—C7—N1	−179.3 (6)	C10—C9—C14—Cl2	178.0 (5)
C2—C1—C7—N1	0.3 (9)	C8—C9—C14—Cl2	−0.6 (9)

### 4-Bromo-2-({(E)-2-[(E)-2,6-dichlorobenzylidene]hydrazin-1-ylidene}methyl)phenol (II) Hydrogen-bond geometry (Å, º)

**Table:**

*D*—H···*A*	*D*—H	H···*A*	*D*···*A*	*D*—H···*A*
O1—H1*O*···N1	0.83 (6)	1.96 (6)	2.655 (8)	141 (7)
